# Effect of Prenatal Protein Malnutrition on Long-Term Potentiation and BDNF Protein Expression in the Rat Entorhinal Cortex after Neocortical and Hippocampal Tetanization

**DOI:** 10.1155/2008/646919

**Published:** 2008-06-30

**Authors:** Alejandro Hernández, Héctor Burgos, Mauricio Mondaca, Rafael Barra, Héctor Núñez, Hernán Pérez, Rubén Soto-Moyano, Walter Sierralta, Victor Fernández, Ricardo Olivares, Luis Valladares

**Affiliations:** ^1^Department of Biology, Faculty of Chemistry and Biology, University of Santiago of Chile, 3363 Avenida Alameda Bernardo O'Higgins, 9170022 Santiago, Chile; ^2^School of Psychology, Las Americas University, 1 Oriente Mall Marina Arauco, 2541362 Viña del Mar, Chile; ^3^Institute of Nutrition and Food Technology (INTA), University of Chile, 5540 Avenida Macul, 7830489 Santiago, Chile; ^4^Montessori Study Center, 2865 Avenida Duble Alméyda, 7750169 Santiago, Chile; ^5^Department of Animal Biological Sciences, Faculty of Veterinary Sciences, University of Chile, 11735 Avenida Santa Rosa, 8820808 Santiago, Chile

## Abstract

Reduction of the protein content from 25 to 8% casein in the diet of pregnant rats results in impaired neocortical long-term potentiation (LTP) of the offspring together with lower visuospatial memory performance. The present study was aimed to investigate whether this type of maternal malnutrition could result in modification of plastic capabilities of the entorhinal cortex (EC) in the adult progeny. Unlike normal eutrophic controls, 55–60-day-old prenatally malnourished rats were unable to develop LTP in the medial EC to tetanizing stimulation delivered to either the ipsilateral occipital cortex or the CA1 hippocampal region. Tetanizing stimulation of CA1 also failed to increase the concentration of brain-derived neurotrophic factor (BDNF) in the EC of malnourished rats. Impaired capacity of the EC of prenatally malnourished rats to develop LTP and to increase BDNF levels during adulthood may be an important factor contributing to deficits in learning performance having adult prenatally malnourished animals.

## 1. INTRODUCTION

Both human and animal studies indicate that maternal protein malnutrition alters various maturational events in the brain resulting in behavioral abnormalities, altered cognitive functioning, and disturbances in learning and memory (for review, see [1]). Alterations extend into the postnatal period and continue into adulthood. For example, on reaching adulthood prenatally malnourished rats on a 6% prenatal/25% postnatal casein diet exhibit learning disturbances, such as deficits in execution of spatial alternation tasks [2] as well as impaired visual discrimination learning [3]. In addition, on reaching adulthood, rats born from 8% case in-restricted mothers showed decreased visuo-spatial memory performance [4].

One of the principal hypotheses in the malnutrition field relates to the issue that decreases in synaptic plasticity may be a critical brain mechanism underlying learning deficits observed as a result of nutritional insults to the developing brain. In this regard, it has been shown that it is difficult to induce and maintain hippocampal [5, 6] and neocortical [4] long-term potentiation (LTP) in brains of prenatally malnourished rats. Whether prenatal malnutrition could affect synaptic plasticity in brain regions other than the hippocampus and cerebral neocortex is unknown at present. The entorhinal cortex (EC) is well situated to play a key role in the bidirectional interactions between the neocortex and the hippocampus, and is thought to be critically involved in the formation of declarative (or explicit) memory—the ability to recollect everyday events and factual knowledge. Potentially, prenatal malnutrition could alter at this level the bidirectional communication between the neocortex and the hippocampus, thereby disturbing some types of cognitive processes. However, the effects of prenatal malnutrition on EC neuroplastic mechanisms have not still been explored. The superficial layers (I–III) of the EC are regarded as “input layers” since terminations of the projections from perirhinal and parahippocampal cortices—the recipients of cortical association areas—occur primarily in these layers [7–11]. Information processed in the hippocampus and subiculum is then returned to the deep layers (V-VI) of the EC via projections from the CA1 field and subiculum [11–14], and these layers in turn project to forebrain structures [15–17]. Accordingly, the deep layers are regarded as “output layers,” and therefore functionally segregated from the superficial layers. Similar to other brain regions, the EC has been shown to express N-methyl-D-aspartate (NMDA) receptor-dependent LTP [18–20] and long-term depression [20–24], both in in vitro and in vivo experimental paradigms.

The present study was aimed to investigate whether mild reduction of the protein content of the diet of pregnant rats can modify plastic capabilities of the EC in vivo. In contrast to severe forms of maternal malnutrition, mild reduction of the casein content in the diet of pregnant rats from 25 to 8%, calorically compensated for by carbohydrates, results in apparently normal in utero development of fetuses, as assessed by normal maternal weight gain during pregnancy and normal body and brain weights of pups at birth [25]. However, this insidious form of protein maternal malnutrition, so-called “hidden” prenatal malnutrition [25], results in altered noradrenergic function in the neocortex of the offspring together with impaired neocortical LTP and lower visuospatial memory performance [4, 26–28]. The present results provide evidence that mild prenatal malnutrition in rats leads to impaired long-term synaptic potentiation together with decreased expression of brain-derived neurotrophic factor (BDNF) in the EC of adult animals; a neurotropin plays a major role in regulating induction and maintenance of LTP [29, 30].

## 2. MATERIALS AND METHODS

### 2.1. Animals and diets

The experimental protocol and animal management were in accordance with the NIH Guide for the Care and Use of Laboratory Animals [31], and was approved by the Committee for the Ethical Use of Experimental Animals, INTA, University of Chile. Female Sprague-Dawley rats were fed isocaloric purified diets containing either normal (25% casein, providing 22.5% protein) or low (8% casein, providing 7.2% protein) amounts of protein. The other components of the purified diets were as follows. (i) Normal diet: carbohydrate, 50.2%; fat, 15.0%; vitamin mix, 1.0%; salt mix, 4.7%; water, 1.7%; cellulose, 4.2%; L-methionine, 0.4%. (ii) Low protein diet: carbohydrate, 66.5%; fat, 15.0%; vitamin mix, 1.0%; salt mix, 4.7%; water, 1.0%; cellulose, 4.2%; L-methionine, 0.4%. Both diets provide about 4.3 Kcal/g. The dietary paradigm was started 1 day after mating and continued throughout pregnancy. The body weight gain of the pregnant mothers was controlled daily. At birth, all pups were weighed and litters were culled to 8 pups (4 males, 4 females). Afterwards, pups born from mothers fed the 7.2% protein diet were fostered to well-nourished dams (22.5% protein diet) giving birth on that day. Pups born from mothers receiving the 22.5% protein diet were also fostered to well-nourished dams in order to equalize among groups other factors that may depend on the rearing conditions (i.e., stress due to cross-fostering). After weaning, at 22 days of age, all pups were fed a standard laboratory diet providing 22.5% protein.

### 2.2. LTP determinations in the medial EC

Experiments were carried out in 16 normal and 16 malnourished rats of 55–60 days of age. Rats were weighed, anesthetized with 1.5 g/kg i.p. urethane, and placed in a stereotaxic apparatus under artificial ventilation. Reinforcement of anesthesia during the experiments was not necessary since surgical procedures and recordings lasted no longer than 3 hours and, in our experience, 1.5 g/kg i.p. urethane induces profound anesthesia lasting more than 6 hours. Animals never regained consciousness and no changes in heart rate in response to stimulation were detected throughout the experiments.

Field responses were evoked in the left medial EC by electrical stimulation of either the ipsilateral occipital cortex or the ipsilateral ventral CA1 hippocampal region, in an alternated fashion. After exposure of the left occipital lobe, electrical stimulation of the occipital cortex and the ventral CA1 hippocampal region was carried out by means of two independent bipolar side-by-side electrodes composed each by two glued, parylen-insulated, 50-*μ*m-diameter tungsten wires with a 0.8-mm tip separation. One stimulating electrode was positioned in the left occipital cortex at coordinates *A* = −5.8, *L* = −3.5, in mm, in such a way that the longer tip penetrated the cortex by 1.0 mm. The other stimulating electrode was advanced to the ventral CA1 region at coordinates *A* = −5.5, *L* = −5.0, *V* = 7.0, in mm. As has pointed out recently, progressively more ventral CA1 regions innervate progressively more medial regions of the medial entorhinal areas [32], which in turn receive more strong visual input through the parahippocampal cortex (postrhinal cortex in the rat [7]).

**Figure 1:**
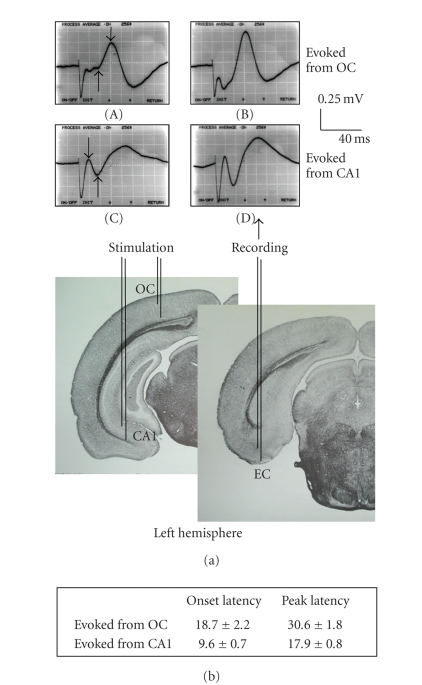
(a) Scheme of two coronal planes of the rat brain illustrating the positions of the stimulating electrodes in the occipital cortex (OC) and ventral CA1 region of the left hemisphere, as well as the location of the recording electrode in the ipsilateral entorhinal cortex EC. In the upper part are shown representative examples of the average of 30 successive responses evoked in the medial EC of one rat by ipsilateral stimulation of the occipital cortex (A and B) or the ventral CA1 hippocampal region (C and D) at 0.2 Hz, obtained before (A and C) and after (B and D) tetanization. Calibration bars are indicated. Upward potential deflection is negative. First and second arrows indicate, respectively, the beginning and the peak of the early negative (A) or early positive (C) wave, which served to calculate peak amplitude or slope (amplitude/time ratio on the nearest sample to the 10% and the 90% level) of the early component. (b) Onset and peak latencies (values are means ± SEM, in millisecond) of the early component of EC responses to test stimulus applied to either the OC or CA1 region before tetanization.

Field responses were recorded from the left medial EC with another bipolar side-by-side electrode (two glued, parylen-insulated, 50-*μ*m-diameter tungsten wires with a 0.8-mm tip separation) positioned at coordinates *A* = −7.5, *L* = −5.0, *V* = 6.5, the longer tip being 0.1-0.2 mm above the ventral brain surface. Configuration and positioning of the recording electrode pair into the EC allowed one tip of the bipolar electrode was into layer II-III and the other tip near layer V. Although bipolar electrode arrangement does not allow performing laminar analysis of potential reversal, it maximizes field recordings corresponding to depolarization of neurons (active inward current or sink) situated near to one electrode tip, while minimizing those produced in distant current generators affecting rather similarly the two electrode tips. Thus, bipolar electrodes seem especially appropriate for focalized recording from laminar cortical structures such as the EC, where differential activation of neurons of layers II-III by neocortical-EC pathways, or layer V by CA1-EC afferents, will create, respectively, superficial and deep current sinks. Rostrocaudal (*A*) and lateral (*L*) coordinates were relative to bregma, while vertical (*V*) coordinates were relative to the cortical surface, all taken from Paxinos and Watson [33]. Figure 1 shows a scheme of two coronal planes of the rat brain illustrating the positions of the stimulating electrodes in the occipital cortex and ventral CA1 region of the left hemisphere, as well as the location of the recording electrode in the ipsilateral EC. Test stimuli, alternately applied to either the occipital cortex (during 2.5 minutes) or the ventral CA1 region (during 2.5 minutes), consisted of 100 microseconds duration square-wave pulses at 0.2 Hz generated by means of a Grass S11 stimulator in conjunction with a Grass SIU-5 stimulus isolation unit and a Grass CCU 1A constant current unit (Astro-Med Inc., West Warwick, RI, USA). Bipolar recording of EC field responses to occipital cortex stimulation consisted of a bigger upward negative wave followed by a downward positive component. Surface negative responses have been already recorded in vivo from the EC of rats during stimulation of the piriform cortex [34]. In turn, EC field responses to CA1 stimulation begin with a marked downward surface positive deflection followed by a late upward surface negative wave of smaller amplitude. In vivo recording of surface positive field responses from the EC of rats during CA1 stimulation has recently been reported [35–37]. Thus, only the negative first-wave of occipital cortex-EC responses and the positive first-wave of CA1-EC responses were measured in the present experiments. Before beginning each experiment, two full input-output series, one for the occipital cortex and the other for CA1, were performed at stimulus intensities of 200–1200 *μ*A. Test stimuli with a stimulation intensity yielding EC responses with first-wave peak amplitude of 50% of the maximum were used for the remainder of the experiment. Thus, test stimuli applied to the occipital cortex were similar in frequency and duration to those applied to CA1, but rather different in intensity. EC responses evoked from the occipital cortex and from the CA1 region were also subjected to a 10-pulse, 30 Hz stimulus in order to test the ability of the response to follow repetitive stimulation. As showed elsewhere [34, 38], polysynaptic components usually fail at frequencies <40–50 Hz, whereas monosynaptic components should follow frequencies near 100 Hz. After a 30-minute stabilization period of alternated occipital cortex and CA1 stimulation with test stimuli, a 2.5-minute control period of EC basal responses (30 averaged responses) evoked from the occipital cortex was recorded, followed by another 2.5-minute control period of EC basal responses (30 averaged responses) evoked from the CA1 region. Thereafter, LTP was induced in the medial EC by applying tetanizing stimulation either to the occipital cortex (8 normal and 8 malnourished rats) or to the ventral CA1 region (8 additional normal and 8 additional malnourished rats). The tetanizing stimulus consisted of three high-frequency trains (100 microseconds square-wave pulses at 312 Hz) of 500 milliseconds duration each, applied every 30 seconds with intensity 50% higher than the respective test stimuli. Such a stimulating protocol has been shown to induce saturating LTP in the EC, at least when activating the EC from the piriform cortex in awake rats, meaning that subsequent application of additional trains fails to induce further increments of field responses [34]. A closely similar stimulating protocol (3 trains of stimuli for 200 milliseconds at 250 Hz with intertrain interval of 30 seconds) applied to the CA1 hippocampal region has been reported to produce reliable LTP induction in the EC of uretanized rats [37].

Recordings were amplified by a Grass P-511 preamplifier (0.8–1000 Hz bandwidth), and displayed and averaged on a Philips PM 3365A digital oscilloscope. They were also digitized at a rate of 10000/second by an A/D converter interfaced to an Acer PC, and stored for retrieval and offline analysis. In all experiments, body temperature and expired CO_2_ were monitored and remained within normal limits. Peak latency and peak amplitude of the early component of averaged field responses were measured using time and voltage cursors provided in the digital oscilloscope. Slope was determined as the amplitude/time ratio on the nearest sample to the 10% and the 90% level between cursors set on the beginning and the peak of the early negative or positive wave (see Figure 1, first and second arrow, resp., in recordings (A and C)). The efficacy of the tetanizing train to potentiate cortical evoked responses was evaluated by measuring both the peak amplitude and the maximal slope increases. The results were similar but the former procedure led to lower variability of means (as revealed by statistical variance), so amplitudes were used for analyses of the experiments.

Two hours after occipital cortex or CA1 tetanization, once the electrophysiological experiments were finished, the animals were sacrificed by decapitation, the brain rapidly removed and weighed, and the left and right ECs dissected out. The average weight of dissected entorhinal area (averaged without taking into consideration left or right hemisphere origin) was 5.77 ± 0.61 for normal rats and 5.30 ± 0.50 for malnourished animals (mean ± SEM). These samples were stored at −80°C before use. Afterwards, the tissues were examined for expression of BDNF protein level by ELISA.

### 2.3. Determination of BDNF in the EC

Whole samples of EC were homogenized in ice-cold lysis buffer, containing 137 mM NaCl, 20 mM Tris-HCl (pH 8.0), 1% Triton X-100, 10% glycerol, and 2 *μ*L/mL protease inhibitor cocktail P8340 (Sigma-Aldrich, St. Louis, MO, USA). The tissue homogenate solutions were centrifuged at 14000 x g for 5 minutes at 4°C. The supernatants were collected and diluted 1/5 in buffer DPBS and then acidified in 1 N HCl. They were then incubated for 15 minutes at room temperature and neutralized with 1 N NaOH until pH 7.6. BDNF was assessed using the E-Max ImmunoAssay system ELISA kit (Promega, Co., Madison, Wis, USA). Briefly, standard 96-well flat-bottom NUNC-immuno maxisorp ELISA plates were incubated overnight at 4°C with a monoclonal anti-BDNF antibody. The plates were blocked by incubation for 1 hour at room temperature (RT) with a 1x block and sample buffer. Serial dilutions of known amounts of BDNF ranging from 0 to 500 pg/mL were performed in duplicate for standard curve determination. Wells containing the standard curves and supernatants of brain tissue homogenates were incubated at room temperature for 2 hours, as specified by the protocol. They were then incubated with a secondary antihuman BDNF polyclonal antibody for 2 hours at room temperature, as specified by the protocol. A species-specific antibody conjugated to horseradish peroxidase was used for tertiary reaction for 1 hour at room temperature following this incubation step. TMB one solution was used to develop color in the wells. This reaction was terminated with 1 N hydrochloric acid at a specific time (10 minutes) at room temperature, and absorbance was then recorded at 450 nm in a microplate reader within 40 minutes of stopping the reaction. The neurotrophin values were determined by comparison with the regression line for BDNF and expressed as pg BDNF/mg wet weight. Using this kit, BDNF can be quantified in the range of 7.8–500 pg/mL.

### 2.4. Statistical analyses

All statistical analyses were performed with GraphPad Instat version 3.00 (GraphPad Software, Inc., San Diego, Calif, USA). For the effect of dietary treatments on body and brain weights, intergroup comparisons were made using unpaired Student's *t*-test. For the analysis of the time-course in LTP studies, a one-way ANOVA was performed for intragroup comparisons followed by Dunnett's multiple comparisons post-hoc test. For analyzing results of BDNF protein expression, intergroup comparisons between normal and malnourished groups were made using unpaired Student's *t*-test, while the effect of tetanization was assessed using nonparametric ANOVA (Kruskal-Wallis test) followed by Dunn's multiple comparisons post-hoc test.

## 3. RESULTS

### 3.1. Effect of dietary treatment on body and brain weights

Body and brain weights measurements revealed that there were no significant differences in body weight gain of pregnant mothers receiving 7.2% or 22.5% protein diet (data not shown). Full data on the effects of this dietary treatment on maternal weight gain during the first, second, and third weeks of pregnancy was published elsewhere [26]. At days 1, 8, and 55–60 of postnatal life, no significant differences in body and brain weights were found between rats born from mothers receiving 7.2% or 22.5% protein diet (Table 1).

### 3.2. LTP in vivo in the medial EC

In rats of 55–60 days of age, bipolar recording of basal EC field responses to occipital cortex stimulation consisted of a prominent upward negative wave followed by a positive component. This is consistent with the arrangement of the side-by-side bipolar electrode located into the EC, where the longer tip is expected to be recording an early superficial sink generated by depolarization of stellate and pyramidal principal neurons within input layers II-III (in relation to rather silent deep layers). In contrast, basal EC responses to CA1 stimulation began with a marked downward surface positive deflection followed by a late surface negative wave, which is consistent with the recording through the shorter tip of an early deep sink resulting from depolarization of pyramidal cells within output layer V. Figure 1(a) shows typical recordings of basal (A and C) and potentiated (B and D) averaged EC field responses evoked by stimulation of the occipital cortex (A and B) or the CA1 region (C and D). In normal eutrophic rats, the onset and peak latencies of the early negative component of basal EC responses evoked from the occipital cortex were 18.7 ± 2.2 and 30.6 ± 1.8 milliseconds, respectively, while the onset and peak latencies of the early positive wave in basal responses evoked from CA1 were 9.6 ± 0.7 and 17.9 ± 0.8 milliseconds (Figure 1(b)). For both type of responses, the differences in latency before and after potentiation were not statistically significant (paired Student's *t*-test, *N* = 8). Shape, latencies, and wave amplitudes of basal field responses evoked in the EC of prenatally malnourished rats, either from the occipital cortex or the CA1 area, did not differ from those of normal eutrophic rats (unpaired Student's *t*-test, *N* = 8 in each group). Frequency testing showed that the early component of the EC potential evoked from the occipital cortex declined rapidly with a stimulus frequency of 30 Hz, thus suggesting a polysynaptic nature of the response. In contrast, the early component of the EC response elicited from CA1 was able to follow 30 Hz stimulation frequency with decreasing amplitude of less than 20%, which is characteristic of monosynaptic responses.

**Figure 2:**
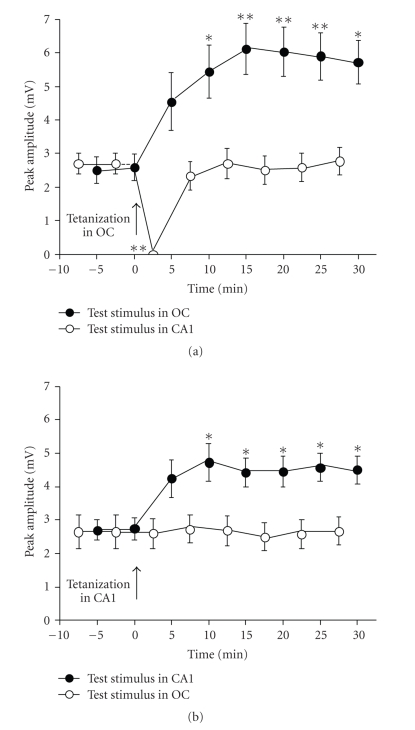
Time-course of LTP induced in the medial entorhinal cortex of 55–60-day-old normal eutrophic rats by applying tetanizing stimulation to the occipital cortex (a) or to the ventral CA1 hippocampal region (b). The arrow indicates time of application of the tetanizing stimulus. *N* = 8 rats in all groups. Values are means ± SEM of peak-to-peak amplitudes, 30 responses averaged per rat. Note the occurrence of homosynaptic, but not heterosynaptic potentiation. One-way ANOVA followed by Dunnett's multiple comparisons test indicated significant intragroup differences in peak-to-peak amplitudes (*P < .05, **P < .01) when comparing post-tetanizing values with the last pretetanizing basal point (at 0 minute), excepting for block 2.5–5 minutes (a), where significant inhibition (**P < .01) occurred.

In normal animals, tetanizing stimulation applied to either the occipital cortex or the CA1 hippocampal region produced a significant increase in peak amplitude of the early component evoked in the ipsilateral medial EC, which remained unchanged throughout the recording period (Figure 2). After tetanizing the occipital cortex, the early negative wave to occipital cortex test stimuli was potentiated to neocortical test stimuli in all blocks over the time-course (ranging from 107 to 136%, Dunnett's multiple comparisons test) excepting for block 2.5–5 minutes, while no significant potentiation to CA1 stimuli was observed in the early component of EC responses (Dunnett's multiple comparisons test); however, a transient but complete inhibition was early observed on block 0–2.5 minutes. After tetanizing the ventral CA1, the early positive wave of EC responses to CA1 test stimuli was potentiated in all blocks over the time-course (ranging from 59 to 72%, Dunnett's multiple comparisons test) excepting for block 2.5–5 minutes, while no significant potentiation to occipital cortex stimulation was observed.

**Figure 3:**
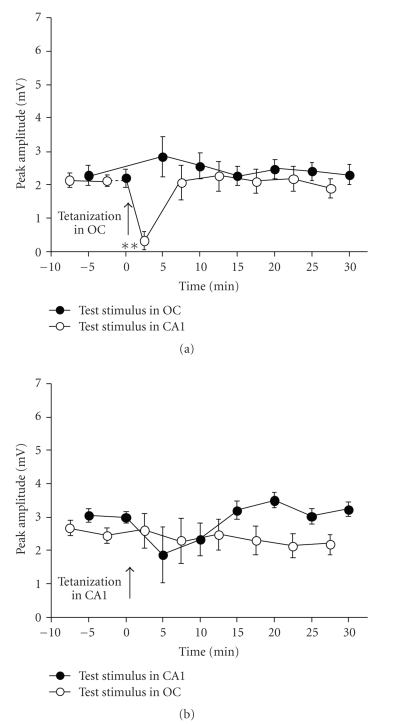
Failure of tetanizing stimulation applied to the occipital cortex (a) or to the ventral CA1 hippocampal region (b) to induce LTP in the medial entorhinal cortex of 55–60-day-old prenatally malnourished rats. The arrow indicates time of application of the tetanizing stimulus. *N* = 8 rats in all groups. Values are means ± SEM of peak-to-peak amplitudes, 30 responses averaged per rat. It can be noted that neither homosynaptic nor heterosynaptic potentiation occurred in the EC of malnourished animals. One-way ANOVA followed by Dunnett's multiple comparisons test indicated no significant intragroup differences in peak-to-peak amplitudes when comparing posttetanizing values with the last pretetanizing basal point (at 0 minute), excepting for block 2.5–5 minutes (a) where significant inhibition (**P < .01) occurred.

In contrast to that occurred in normal controls, no significant increase of the early component of EC field responses evoked from the occipital cortex or from the CA1 region (*P* > .05 for all blocks, Dunnett's multiple comparisons test) was observed in malnourished animals after applying neocortical or hippocampal tetanizing stimulation (Figure 3).

### 3.3. BDNF expression in the EC

Serial dilutions of known amounts of BDNF ranging from 0 to 500 pg/mL allowed to determine a standard curve demonstrating a direct relationship between optical density and BDNF concentration (*r* ^2^ = 0.9106).

Table 2 shows that application of tetanizing stimulation to the left CA1 region in normal rats resulted in a significant increase of BDNF concentration in the ipsilateral EC (*P* < .05, Dunn's multiple comparisons test) two hours after tetanization, while application of tetanizing stimuli to the left occipital cortex did not significantly modify the BDNF level in the ipsilateral EC. In contrast, tetanizing stimulation applied to either the occipital cortex or the CA1 hippocampal region in malnourished rats was ineffective in modifying the BDNF concentration in the ipsilateral EC. Table 2 also shows that on days 55–60 of postnatal life, malnourished rats exhibited significant lower concentration of BDNF protein in the right medial EC (corresponding to the nonstimulated cerebral hemisphere) than that observed in normal animals of same ages (*P* < .05, unpaired Student's *t*-test).

## 4. DISCUSSION

Mild reduction of the protein content of the maternal diet of pregnant rats did not significantly alter body and brain weights of pups at birth, indicating that protein deficiency in the 7.2% protein group was masked by caloric compensation with carbohydrates, leading to apparently normal fetal development as assessed by body and brain weights at birth. A similar result has been reported elsewhere [25, 39]. As discussed by others [25, 39], fetal growth retardation and reductions in brain weight after prenatal malnutrition are only produced by severe protein restriction, that is, reduction of the protein content of the maternal diet to less than 6%.

The foregoing electrophysiological data show that the medial EC of normal eutrophic rats can develop LTP in vivo to tetanization of both the occipital cortex and the CA1 hippocampal region. This is consistent with previous data showing that the EC could express LTP to tetanizing stimulation of some cortical and hippocampal regions in in vivo conditions. For example, Chapman and Racine [34] have reported a surface negative response that could be evoked in vivo in the EC of rats by stimulation of the piriform cortex, and that these responses undergo LTP to high-frequency stimulation of the piriform cortex. However, Ivanco and Racine [35] found that stimulation of the motor cortex failed to elicit EC responses. On the other hand, surface positive field responses have been elicited in vivo in the EC of rats by stimulation of CA1 [35–37]. In all these studies, the early positive component of the EC response supported LTP to high frequency stimulation.

The present results also suggest that the early negative response evoked in the entorhinal cortex (EC) by occipital cortex stimulation is apparently polysynaptic, since it had slow onset and peak latencies and it was very sensitive to 30 Hz stimuli. As reported previously, these inputs are synaptically relayed within the perirhinal and/or parahippocampal cortices before to reach the superficial layers of the EC [7–11]. Polysynaptic LTP often involves local circuits within the recipient brain region, but sometimes is synaptically relayed by brain intermediate regions that are more distant from the recipient zone thus involving long axonmediated connections. Regarding the present results, it is not possible to directly known if potentiation occurred in neurons of the final recipient entorhinal region or in intermediate perirhinal neurons relaying the response (or in both) and, therefore, the site of LTP occurrence remains rather unresolved. In contrast, the early positive response evoked in the entorhinal cortex (EC) by CA1 stimulation is apparently monosynaptic since it had shorter onset and peak latencies and followed a stimulus frequency of 30 Hz without showing a significant amplitude decrease. As mentioned previously, direct projections from the CA1 field to the deep V-VI layers of the EC have already been reported [15–17].

The fact that tetanization of the occipital cortex or the CA1 region only potentiates the responses driven by the tetanized region confirms that the early component of EC responses to either occipital cortex or CA1 stimulation represents the activation of two distinct set of neurons (presumably located in superficial layers II-III and deep layer V, resp.). Nevertheless, prenatally malnourished adult rats were unable to develop LTP in the medial EC, at least when submitted to a similar tetanizing stimulation protocol to that applied to the occipital neocortex or the ventral CA1 region in normal eutrophic animals, thus suggesting that mild prenatal malnutrition impairs some neural substrate involved in the generation and/or maintenance of EC plasticity. Previous reports have shown that it is difficult to induce and maintain hippocampal [5, 6] and neocortical [4] LTP in brains of prenatally malnourished rats, but the underlying cellular/molecular mechanisms are still unresolved. Reduced plastic response in the hippocampus of prenatally malnourished rats seems to be related to significant increases in GABAergic inhibition in the dentate gyrus [1, 40], while in neocortex it may be correlated with decreased noradrenaline release due to enhanced *α*_2C_ adrenoceptor expression [4, 28]. However, the effect of prenatal malnutrition on neuroplastic mechanisms operating in the EC had not yet been explored.

Interestingly, the medial EC of normal eutrophic rats showed increased BDNF concentration two hours after delivering tetanizing stimulation to the ipsilateral CA1, whereas the same stimulatory protocol failed in modifying the BDNF level in the EC of prenatally malnourished rats. As has previously been reported, high-frequency stimulation inducing LTP evokes significant increases in BDNF mRNA expression [41–44] and BDNF release [45] in the hippocampus, although changes in hippocampal BDNF protein levels after LTP induction, have not still been evaluated. In addition, released BDNF activates distinct mechanisms to regulate the induction, early maintenance, and late maintenance phases of hippocampal LTP [29, 30]. Curiously, LTP induced by unilateral perforant path stimulation seems to produce bilateral induction of BDNF mRNA, although limited to the dentate gyrus [42–46]. A more detailed study addressing this aspect was carried out by Bramham et al. [47], who demonstrated that unilateral LTP induction in the dentate gyrus of awake rats led to highly selective ipsilateral (trkB and NT-3 mRNA) or bilateral (trkC, BDNF, and nerve growth factor mRNA) increases in gene expression, indicating that LTP triggers an interhemispheric communication manifested as selective, bilateral increases in gene expression at multiple sites in the hippocampal network. Whether changes in BDNF concentration occurred bilaterally in the medial EC after unilateral tetanization of CA1 could not be assessed in the present study, because of the BDNF level in the EC of the nonstimulated right side served as control for the BDNF value obtained in the EC of the left stimulated side. Nevertheless, despite the inexistence of a proper control taken from nontetanized rats, the present data show that there was a significant difference in BDNF concentration when values from right (nonstimulated) ECs were compared with those from left (stimulated) ECs in normal eutrophic rats, whereas such a difference was not present in entorhinal tissue from malnourished animals. Failure of tetanizing stimulus in modifying BDNF levels in the ipsilateral EC of prenatally malnourished rats (so-called “instructive mechanisms” [29, 30], those initiated in response to high-frequency stimulation and required for subsequent development of LTP) clearly match the inability of the medial EC to induce LTP in malnourished animals, but caution must be exercised regarding this issue because this observation reveals a correlational but not causal relationship. Additionally, malnourished animals had significantly lower concentration of BDNF in the right EC (supposedly “basal” levels in the nonstimulated side) than normal ones, thus suggesting a possible additional deficit in “permissive mechanisms” of BDNF (those that make synapses competent for LTP [29, 30]).

Why application of tetanizing stimulation to the occipital cortex of normal eutrophic rats resulted in potentiation of ipsilateral evoked EC responses, but not in increased BDNF concentration in the same EC, is presently unclear. One plausible explanation is that occipital cortex tetanization really increased BDNF expression but solely in some restricted layers of the EC and therefore they were not detected by staining the whole EC. Alternatively, it is possible that this type of polysynaptic LTP did not actually lead to increased BDNF expression. Then, this negative result in the occipital cortex-EC pathway should be interpreted with caution as the analysis performed is quite preliminary. Comparable tetanizing stimulation of the occipital cortex in malnourished rats did not induce either LTP or BDNF protein enhancement in the ipsilateral EC. Furthermore, high-frequency stimulation of the occipital cortex gave rise to a short period (about 5 minutes) of depression (or even irresponsiveness) of ipsilateral EC neurons to CA1 stimulation, both in normal and malnourished animals (see Figures 2(a) and 3(a)). Whether this transient presumably intra-EC inhibitory activity resulted from a feedforward inhibitory mechanism [48] or from a feedback mechanism triggered by the returning CA1 output into deep layers of the EC (see Craig and Commins [36]) remains to be determined. Also, the possibility that the complete loss of the CA1-evoked EC response after tetanizing the OC could be the result of generating local spreading depression cannot be discarded. In this regard, spreading depression-like episodies that were confined to the first 5 minutes after tetanizing the perforant path-granule cell pathway have been reported in anesthetized rats [49].

In summary, the present data show that mild prenatal protein malnutrition resulted in impaired ability of the EC to undergo LTP and to increase BDNF levels in response to tetanizing stimulation of the ipsilateral ventral CA1 hippocampal region during postnatal life. On the basis that EC is part of a circuit underlying networked representations of previous experiences via bidirectional connections between the neocortex with the hippocampus, impaired EC plasticity may be an important factor contributing to deficits in explicit learning having adult, prenatally malnourished animals.

## Figures and Tables

**Table 1:** Body and brain weights of normal and prenatally malnourished rats. Values are means ± SEM. *N* = 16 rats in each group. No statistically significant differences (NS) were found when comparing body and brain weights of normal and malnourished groups of same ages (unpaired Student's *t*-test).

	Body weight (g)			Brain weight (mg)
Age	Day 1	Day 8	Day 55	Day 55–60
Normal	7.3 ± 0.07	18.9 ± 0.37	235 ± 10	1322.0 ± 16.1
Malnourished	7.2 ± 0.09	19.1 ± 0.45	229 ± 12	1318.6 ± 15.7
*P*	NS	NS	NS	NS

**Table 2:** Changes in BDNF expression (pg/mg wet tissue) in the left entorhinal cortex (EC) of 55–60-day-old normal and prenatally malnourished rats two hours after applying ipsilateral tetanizing stimulation to the occipital cortex (OC) or the CA1 hippocampal region, as compared to BDNF levels in the right EC. Values are means ± SEM. The number of samples in each group is shown in parentheses. BDNF concentrations in right EC samples after tetanizing the left OC or left CA1 did not significantly differ between them, and were therefore pooled. Comparisons of BDNF levels between normal and malnourished groups were made using unpaired Student's *t*-test, and *P*_NC_ is the probability level for comparisons related to the nutritional condition (NS = not significant). Comparisons between basal BDNF levels (right EC) with those obtained after OC or CA1 tetanization (left EC) were made using nonparametric ANOVA (Kruskal-Wallis test) followed by Dunn's multiple comparisons post-hoc test, and *P*_T_ is the probability level for comparisons between right and left EC samples (different superscripts indicate a significant difference, *P* < .05; NS = not significant).

	Pooled OC + CA1 tetanization	OC tetanization	CA1 tetanization	*P*_T_
	(right EC)	(left EC)	(left EC)	
Normal	15.7 ± 3.5^a^(8)	14.4 ± 2.6^a^(4)	25.1 ± 2.2^b^(4)	<0.05
Malnourished	9.5 ± 0.82 (8)	11.4 ± 2.0 (4)	8.1 ± 1.6 (4)	NS
*P*_NC_	<0.05	NS	<0.001	
